# Associations between genetic variants of *KIF5B*, *FMN1*, and *MGAT3* in the cadherin pathway and pancreatic cancer risk

**DOI:** 10.1002/cam4.3603

**Published:** 2020-11-16

**Authors:** Lingling Zhao, Hongliang Liu, Sheng Luo, Patricia G. Moorman, Kyle M. Walsh, Wei Li, Qingyi Wei

**Affiliations:** ^1^ Cancer Center The First Hospital of Jilin University Changchun Jilin China; ^2^ Duke Cancer Institute Duke University Medical Center Durham NC USA; ^3^ Department of Medicine Duke University School of Medicine Durham NC USA; ^4^ Department of Biostatistics and Bioinformatics Duke University School of Medicine Durham NC USA; ^5^ Department of Neurosurgery Duke University School of Medicine Durham NC USA; ^6^ Department of Family Medicine and Community Health Duke University Medical Center NC USA; ^7^ Department of Population Health Sciences Duke University School of Medicine Durham NC USA

**Keywords:** cadherin pathway, pancreatic cancer, risk analysis, single‐nucleotide polymorphism

## Abstract

Because the cadherin‐mediated signaling pathway promotes cancer progression, we assessed associations between genetic variants in 109 cadherin‐related genes and risk of pancreatic cancer (PanC) by using genotyping data from publically available genome‐wide association studies (GWAS) datasets comprising 15,423 individuals of European ancestry. After initial single‐locus analyses and subsequent meta‐analysis with multiple testing correction for 29,963 single‐nucleotide polymorphisms (SNPs), 11 SNPs remained statistically significant (*p* < 0.05). In the stepwise logistic regression analysis, three independent PanC risk‐associated SNPs (*KIF5B* rs211304 C > G, *FMN1* rs117648907 C > T, and *MGAT3* rs34943118 T > C) remained statistically significant (*p* < 0.05), with odds ratios of 0.89 (95% confidence interval = 0.82–0.95 and *p* = 6.93 × 10^−4^), 1.33 (1.13–1.56 and 2.11 × 10^−4^), and 1.11 (1.05–1.17 and 8.10 × 10^−5^), respectively. Combined analysis of unfavorable genotypes of these three independent SNPs showed an upward trend in the genotype‐risk association (*p*_trend_ < 0.001). Expression quantitative trait loci analyses indicated that the rs211304 G and rs34943118 C alleles were associated with increased mRNA expression levels of *KIF5B* and *MGAT3*, respectively (all *p* < 0.05). Additional bioinformatics prediction suggested that these three SNPs may affect enhancer histone marks that likely have an epigenetic effect on the genes. Our findings provide biological clues for these PanC risk‐associated SNPs in cadherin‐related genes in European ancestry populations, possibly by regulating the expression of the affected genes. However, our findings need to be validated in additional population, molecular and mechanistic investigations.

## Graphical abstract

We assessed associations between genetic variants in 109 cadherin‐related genes and pancreatic cancer risk by using genotyping data from two available genome‐wide association study datasets for 8477 cases and 6946 controls of European ancestry. We identified three novel PanC risk‐associated SNPs, *KIF5B* rs211304 C > G, *FMN1* rs117648907 C > T and *MGAT3* rs34943118 T > C. We also demonstrated potential functions of these risk loci, especially for the SNP rs211304 and rs34943118, to be associated with mRNA expression levels of *KIF5B* and *MGAT3*, respectively.

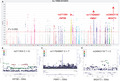

## 1 INTRODUCTION

Pancreatic cancer (PanC) is one of the most fatal cancers characterized by early metastasis and high mortality. Globally, PanC is the seventh leading cause of cancer deaths.^1^ In the United States, an estimated 57,600 and 47,050 people will be diagnosed with and die of PanC, respectively, in 2020,^2^ with a projection to become the second common cause of cancer death by 2030.^3^ To date, PanC remains a major public health burden in the United States.^4^ Therefore, early detection and prevention is urgently needed by identifying those with high risk of PanC.

Several risk factors are associated with PanC, including age, sex, race, smoking, alcohol, obesity, dietary factors, diabetes mellitus, family history of PanC, chronic pancreatitis, and certain single‐nucleotide polymorphisms (SNPs).^1^ Over the last decade, thousands of genome‐wide association studies (GWASs)‐based significant (i.e., *p* < 5.0 × 10^−8^) genetic variants have been found to be related to the risk for complex human disorders, including PanC.^5^, ^6^, ^7^, ^8^, ^9^ Nevertheless, up to now, only a small fraction of these statistical associations have been found to be functionally relevant at the molecular level.^10^ Furthermore, in these hypothesis‐free GWAS, many causal genetic variants affecting disease risk may be overlooked because of the stringent *p*‐value cutoffs due to the need for multiple testing corrections as a consequence of the large number of SNPs to have been tested. An alternative approach to deal with this daunting hypothesis‐free issue is to use biological pathway‐related gene‐set analysis, an important and useful hypothesis‐driven approach that leverages existing GWAS datasets to detect functionally plausible disease‐associated SNPs.^10^, ^11^

The cadherin signaling pathway encompasses a large superfamily of membrane proteins that regulate calcium‐dependent cell–cell adhesion and tissue morphogenesis.^12^ Studies have shown that changes in cadherin‐mediated signaling pathways promote cancer progression.^13^, ^14^ For example, one study found that aberration in expression of cadherin 1 was involved in pancreatic duct cell carcinogenesis,^13^ while another study reported that beta‐1,4‐mannosyl‐glycoprotein 4‐beta‐N‐acetylglucosaminyltransferase (MGAT3) was associated with posttranslational modification of cadherin 1.^15^ Other studies reported that the activity of MGAT3 was significantly increased in PanC^16^ and that cadherin 2 played an important part in facilitating the malignant phenotype in PanC.^17^ Furthermore, the upregulation of catenin delta‐1 was found to be related to proliferation of PanC cells and an aggressive phenotype of PanC,^18^ and the kinesin family member 5B (KIF5B) participated in transporting cadherin‐catenin complexes to adhesion junctions.^19^ In addition, other cadherin signaling members (e.g., catenin beta 1 and cell division cycle 42) have also been found to be associated with pancreas tumorigenesis.^20^, ^21^

In the current study, we hypothesize that genetic variants in cadherin‐related genes are associated with PanC risk. We tested this hypothesis by using publicly available GWAS data from the Pancreatic Cancer Cohort Consortium (PanScan) and the Pancreatic Cancer Case‐Control Consortium (PanC4) in populations of European ancestry.

## 2 METHODS AND MATERIALS

### 2.1 Study participants

In the current analysis, we used the existing genotyping data from the PanScan (dbGaP#: phs000206.v5.p3) and the PanC4 (dbGaP#: phs000648.v1.p1) studies in populations of European ancestry as described previously (Figure S1).^5^, ^6^, ^7^, ^8^, ^22^, ^23^ The PanScan study included PanScan I, PanScan II, and PanScan III (1760 cases/1780 controls, 1457 cases/1666 controls, and 1538 cases/0 controls, respectively).^5^, ^6^, ^7^ Because PanScan III comprised only cases, PanScan II and PanScan III were merged and analyzed jointly, hereafter referred to as PanScan II/III. The PanC4 study included 3722 cases and 3500 controls from nine case‐control studies.^8^, ^22^, ^23^ As illustrated in Table S1, 8477 cases and 6946 controls were included in the analysis. Written informed consent was obtained from each participant in the original studies. Approval for the current study protocol was obtained from the Duke University Health System Institutional Review Board (Pro00054575).

### 2.2 Gene and SNP selection

**Figure 1:**
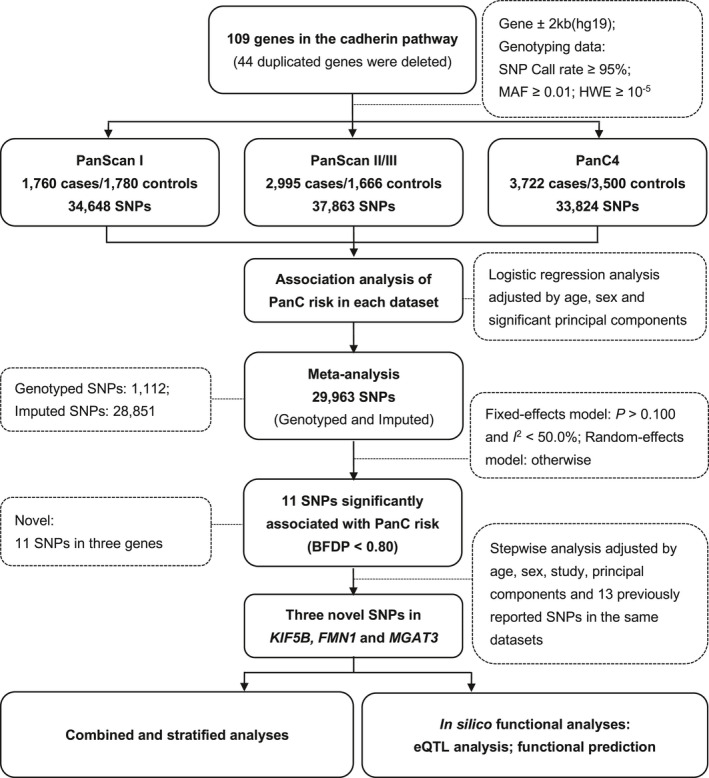
Study flowchart. BFDP, Bayesian false discovery probability; eQTL, expression quantitative trait loci; HWE, Hardy–Weinberg equilibrium; MAF, minor allele frequency; PanC, pancreatic cancer; SNP, single‐nucleotide polymorphism.

The cadherin pathway‐related gene‐set was selected from the Molecular Signatures Database (MSigDB, v7.0)^24^ and the PathCards^25^ by using the keyword “cadherin.” After deleting 44 duplicated genes, a total of 109 candidate genes remained for further analyses (Table S2). Gene regions were defined and obtained from the Genome Reference Consortium Human Build 37 (GRCh37)/hg19 database. Imputation was conducted for each dataset (i.e., PanScan I, PanScan II, PanScan III, and panC4 studies) separately with IMPUTE2 by using the genotyping data of SNPs within ±500 kb flanking regions of these candidate genes from those GWASs and the 1000 Genomes Project database (phase 3 release v5).^26^ Imputed and genotyped SNPs mapped within ±2 kb flanking regions of each gene were extracted in each dataset separately. As there were no controls for the PanScan III dataset, PanScan II and PanScan III extraction data for the cases were merged into PanScan II/III for further analyses. A meta‐analysis was further performed for all datasets with the following criteria of SNP exclusion: Hardy–Weinberg equilibrium (HWE) *p*‐value of <1 × 10^−5^, an imputation info score of <0.5, a minor allele frequency (MAF) of <0.01, and a SNP call rate of <95% (Figure 1; Figure S2).

### 2.3 Statistical analysis

Single‐locus analysis was performed by using multivariable logistic regression model with PLINK 1.9^27^ to assess associations between SNPs and PanC risk with adjustment for age, sex, and top significant principal components (PCs) in each dataset (PanScan I, PanSan II/III, and PanC4). Significant PCs were predetermined in logistic regression models, leading to five significant PCs in each of PanScan I and PanSan II/III as well as seven significant PCs in PanC4. Odds ratio (OR) and 95% confidence interval (CI) were estimated by using an unconditional logistic regression analysis to assess the strenngth of associations between SNPs and risk of pancreatic cancer. Inverse variance weighted meta‐analysis was conducted to combine the association results from the three studies by using PLINK 1.9.^28^ A fixed‐effects model was adopted when the Cochran's *Q* test *p*‐value was >0.100 and the *I* ^2^ was <50.0%.^29^ Otherwise, a random‐effects model was applied.

For multiple testing correction, the Bayesian false discovery probability (BFDP) approach with a cutoff value of 0.80 was applied,^30^ when SNPs failed to pass false discovery rate (FDR), since most of the SNPs under investigation were in high linkage disequilibrium (LD) as a result of imputation. A prior probability of 0.01 was assigned to detect an upper bound of 3.0 for an association with minor alleles or variant genotypes of the SNPs with a *p*‐value of <0.050. Stepwise logistic regression analysis was performed with adjustment for age, sex, study, and the top five significant PCs (*p* < 0.001) (Table S3), as well as the 13 previously reported SNPs from the same datasets to identify independent SNPs for PanC risk (Table S4). Conditional and joint (COJO) analyses were also performed by using Genome‐wide Complex Trait Analysis (GCTA) software to identify independent SNPs in the current study.^31^ The gene‐based test was conducted by using the VEGAS (versatile gene‐based association study) approach integrated in the VEGAS2 program.^32^, ^33^

In the combined unfavorable genotype analysis, cumulative effects of the risk‐associated SNPs were estimated by the number of unfavorable genotypes (NUG). All individuals were divided into two groups: the low‐risk group, with an NUG of zero to one and the high‐risk group, with an NUG of two to three. Furthermore, LD was also calculated by using data from 358 European individuals from the 1000 Genomes Project with Haploview v4.1,^34^ in which the EM method was used to estimate the maximum‐likelihood values of the four gamete frequencies. Regional association plot were generated with LocusZoom^35^ and Manhattan plots were generated with Haploview v4.1.^34^ Other statistical analyses were conducted with SAS software v9.4 and R software v 3.6.2, unless otherwise specified.

### 2.4 *In* *silico* functional prediction and eQTL analysis

Expression quantitative trait loci (eQTL) analyses were conducted with data from two publicly available databases: data of lymphoblastoid cells from 373 European samples were obtained from the 1000 Genomes Project,^36^ and data of 305 normal pancreas, other normal tissue samples and 607 whole blood samples were obtained from the Genotype‐Tissue Expression (GTEx) Project (v8).^37^ The colocalization analysis of the GWAS and eQTL signals was performed by using the Coloc package.^38^ We used the ENCODE^39^ data integrated in the UCSC Genome Browser, RegulomeDB,^40^ SNPinfo,^41^ and HaploReg v4.1^42^ to predict the functions of PanC risk‐associated SNPs. The transcription factor‐biding motifs were predicted by using the Predicting Regulatory Functional Effect by Approximate *p*‐value Estimation (PERFECTOS‐APE)^43^ and the PROMO online tools.^44^

## 3 RESULTS

### 3.1 Association analysis

**Figure 2:**
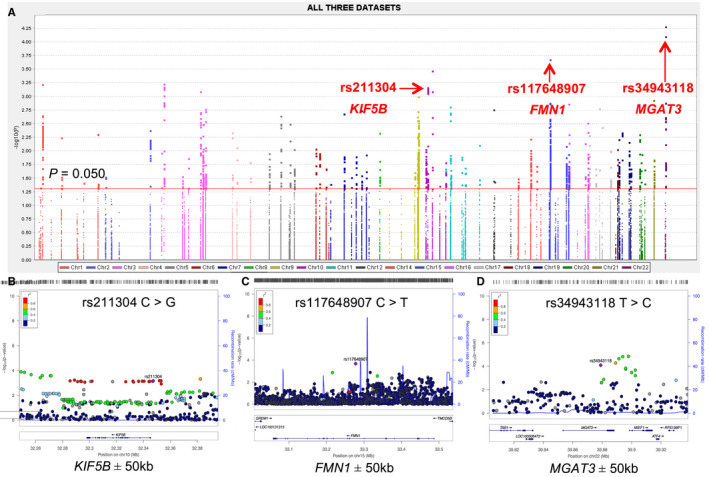
Screening for SNPs in cadherin‐related genes (a) Manhattan plot of the association results of 29,963 SNPs and PanC risk in the meta‐analysis of the three datasets. The horizontal red line represents*p*‐value of 0.050. The three SNPs (i.e., rs211304/rs117648907/rs34943118) shown in the red bold are the novel findings in the current study. Regional association plots of the SNPs within regions of ±50 kb of (b)*KIF5B*rs211304, (c)*FMN1*rs117648907, and (d)*MGAT3*rs34943118. PanC, pancreatic cancer; SNP, single‐nucleotide polymorphism.

The flowchart of this study is illustrated in Figure 1. The basic characteristics of 15,423 individuals of European ancestry from the PanScan and PanC4 datasets have been described elsewhere.^45^ After imputation and quality control, we included 34,648 (PanScan I), 37,863 (PanScan II/III), and 33,824 (PanC4) SNPs for further analysis. We performed a single‐locus analysis to determine the associations between SNPs and PanC risk with adjustment for age, sex, and the significant PCs for each of these three genotyping datasets. Overall, there were 1362 (PanScan I), 1642 (PanScan II/III), and 1884 (PanC4) SNPs found to be significant (*p* < 0.050; Figure S3). After excluding SNPs with an HWE *p*‐value <1 × 10^−5^, an imputation info score <0.5, an MAF < 0.01, and a call rate <95%, the subsequent meta‐analysis included 29,963 SNPs (1112 genotyped and 28,851 imputed) that all three datasets had in common, 1502 of which remained significantly associated with PanC risk (*p* < 0.050). Although none SNP passed the FDR cutoff value (<0.20; Table 1) for multiple test correction, 11 SNPs remained associated with risk of PanC (Table 1) with correction by BFDP < 0.80. To further identify independent SNP associations, we evaluated these newly identified 11 SNPs by using the multivariable stepwise logistic regression analysis and the COJO analysis. As a result, three novel SNPs (i.e., *KIF5B* rs211304 C > G, *FMN1* rs117648907 C > T, and *MGAT3* rs34943118 T > C) remained significantly associated with risk of PanC in the presence of the 13 previously published SNPs (Figure 2a–d; Tables S4–S5). Specifically, as summarized in Table 2, the *KIF5B* rs211304 G allele was associated with a reduced PanC risk (OR = 0.89, 95% CI = 0.82–0.95, *p* = 6.93 × 10^−4^), while the *FMN1* rs117648907 T allele and *MGAT3* rs34943118 C allele were associated with an increased PanC risk (OR = 1.33, 95% CI = 1.13–1.56, *p* = 2.11 × 10^−4^ and OR = 1.11, 95% CI = 1.05–1.17, *p* = 8.10 × 10^−5^) in the final meta‐analysis, none of which exhibited statistically significant heterogeneity. We then conducted the gene‐based test with the VEGAS method and found nine (*RHOA*, *MGAT3*, *WASF2*, *MAPRE1*, *KIF5B*, *RAPGEF1*, *AQP5*, *FMN1*, and *GIT1*) of the 109 cadherin‐related genes with an empirical *p* < 0.05 (Table S6). Interestingly, *MGAT3* rs2017711 in high LD (*r* ^2^ = 0.64, Figure S4) with *MGAT3* rs34943118, *KIF5B* rs211304, and *FMN1* rs117648907 identified in the current study were estimated as the best‐SNPs of their corresponding genes with *p*‐value of 5.27 × 10^−5^, 6.93 × 10^−4^, and 2.10 × 10^−4^, respectively (Table S6).

**Table 1:** Associations between 11 novel SNPs in cadherin‐related genes and PanC risk with correction for multiple testing by BFDP < 0.80

SNP	Chr	Position	Gene	Allele ^a^	MAF1^b^	MAF2^b^	MAF3^b^	Info ^c^	OR (95% CI)^d^	*p* ^e^	FDR	BFDP^f^	Functional consequence
rs34943118	22	39878998	*MGAT3*	T/C	0.24	0.24	0.25	0.9	1.11 (1.05–1.17)	8.10 × 10^−^^5^	0.75	0.53	Intron variant
rs117648907	15	33277710	*FMN1*	C/T	0.02	0.02	0.02	1.0	1.33 (1.13–1.56)	2.11 × 10^−4^	0.75	0.63	Intron variant
rs2017711	22	39889080	*MGAT3*	G/A	0.20	0.20	0.21	1.0	1.12 (1.06–1.19)	5.27 × 10^−5^	0.75	0.69	None
rs211304	10	32347322	*KIF5B*	C/G	0.12	0.12	0.13	1.0	0.89 (0.82–0.95)	6.93 × 10^−4^	0.75	0.79	2 KB upstream variant
rs211393	10	32301899	*KIF5B*	G/A	0.12	0.12	0.13	1.0	0.89 (0.82–0.95)	7.31 × 10^−4^	0.75	0.79	Intron variant
rs211284	10	32332232	*KIF5B*	G/A	0.12	0.12	0.13	1.0	0.89 (0.82–0.95)	7.37 × 10^−4^	0.75	0.79	Intron variant
rs211293	10	32339115	*KIF5B*	A/G	0.12	0.12	0.13	1.0	0.89 (0.82–0.95)	7.37 × 10^−4^	0.75	0.79	Intron variant
rs211294	10	32340472	*KIF5B*	T/G	0.12	0.12	0.13	1.0	0.89 (0.82–0.95)	7.37 × 10^−4^	0.75	0.79	Intron variant
rs211303	10	32346927	*KIF5B*	T/C	0.12	0.12	0.13	1.0	0.89 (0.82–0.95)	7.37 × 10^−4^	0.75	0.79	2 KB upstream variant
rs211299	10	32344747	*KIF5B*	G/A	0.12	0.12	0.13	1.0	0.89 (0.82–0.95)	7.42 × 10^−4^	0.75	0.79	Intron variant
rs211290	10	32336397	*KIF5B*	G/A	0.12	0.12	0.13	1.0	0.89 (0.82–0.95)	7.49 × 10^−4^	0.75	0.79	Intron varia

Abbreviations: BFDP, Bayesian false‐discovery probability; Chr, chromosome; CI, confidence interval; FDR, false discovery rate; MAF, minor allele frequency; OR, odds ratio; PanC, pancreatic cancer; SNP, single‐nucleotide polymorphism.

^a^ Reference allele/effect (minor) allele.

^b^ MAF1 from PanScan I dataset; MAF2 from PanScan II/III dataset; MAF3 from PanC4 dataset.

^c^ Info score from PanScan I, II, III, and PanC4 datasets. Each SNP has the same imputation info score in PanScan I, II, III, and PanC4 datasets.

^d^ Fixed‐effects model: *Q* test *p* > 0.100 and *I* ^2^ < 50.0%; otherwise: random‐effects model.

^e^ *p*‐value from the meta‐analysis of the three datasets.

^f^ BFDP approach with a prior probability of 0.01 and an upper bound of effect size (OR) of 3.0.

**Table 2:** Associations between the three independently associated SNPs and PanC risk in the three PanC genotyping datasets

SNP	Allele^a^	Location	Gene	PanScan I		PanScan II/III		PanC4		All GWAS datasets		Heterogeneity^b^		
				1760 cases/1780 controls		2995 cases/1666 controls		3722 cases/3500 controls		8477 cases/6946 controls				
				OR (95% CI)	*p*	OR (95% CI)	*p*	OR (95% CI)	*p*	OR (95% CI)	*p*		*Q*	*I* ^2^(%)
rs211304	C/G	10p11.22	*KIF5B*	0.81 (0.70–0.94)	0.005	0.94 (0.83–1.07)	0.372	0.89 (0.80–0.98)	0.024	0.89(0.82–0.95)	6.93 × 10^−4^		0.315	13.56
rs117648907	C/T	15q13.3	*FMN1*	1.29 (0.93–1.79)	0.130	1.16 (0.87–1.54)	0.309	1.45 (1.17–1.78)	5.52 × 10^−4^	1.33(1.13–1.56)	2.11 × 10^−4^		0.468	0
rs34943118	T/C	22q13.1	*MGAT3*	1.18 (1.06–1.31)	0.003	1.10 (1.00–1.21)	0.061	1.09 (1.01–1.17)	0.029	1.11(1.05–1.17)	8.10 × 10^−5^		0.478	0

Abbreviations: CI, confidence interval; GWAS, genome‐wide association study; OR, odds ratio; PanC, pancreatic cancer; SNP, single‐nucleotide polymorphism.

^a^ Reference allele/effect allele.

^b^ Heterogeneity assessed by *Q* test or *I^2^*: fixed‐effects model: *Q* test *p* > 0.100 and *I^2^* < 50.0%; otherwise random‐effects model.

### 3.2 Combined and stratified analyses

As shown in Table 3, in the genotype effect analysis, the three novel SNPs demonstrated significant associations with PanC risk (*p*_trend_ = 0.001, <0.001, and <0.001 for *KIF5B* rs211304 C > G, *FMN1* rs117648907 C > T, and *MGAT3* rs34943118 T > C, respectively). The unfavorable genotypes of rs211304 CC, rs117648907 CT+TT, and rs34943118 CC were combined into a genetic risk score (GRS) to evaluate their cumulative effect on PanC risk. The results indicated a dose‐response manner between NUGs and PanC risk with adjustment for available covariates (*p*_trend_ < 0.001, Table 3). All individuals were divided into a low‐GRS group, with an NUG of zero‐one and a high‐GRS group, with an NUG of two‐three. Compared with the low‐GRS group, the high‐GRS group had an increased PanC risk (OR = 1.30, 95% CI = 1.16–1.46, *p* < 0.001, Table 3). In addition, we created a GRS by combining these three novel SNPs and all 13 SNPs previously reported in the same model and found that the joint‐effect on risk of PanC increased as the GRS increased (*p*_trend_ < 0.001, Table S7). Compared with the low‐GRS group (two‐10 NUGs), the high‐GRS group (11–14 NUGs) had an increased PanC risk (OR = 1.46, 95% CI = 1.29–1.64, *p* < 0.001, Table S7).

**Table 3:** Combined analysis of the three independent SNPs and PanC risk in the three PanC genotyping datasets

Genotype	Case (%)	Control (%)	OR (95% CI)^a^	*p* ^a^
*KIF5B* rs211304				
CC	6657 (78.53)	5293 (76.20)	1.00	—
CG	1719 (20.28)	1555 (22.39)	0.88 (0.81–0.95)	0.001
GG	101 (1.19)	98 (1.41)	0.83 (0.63–1.10)	0.199
Trend test				0.001
Dominant model				
CC	6657 (78.53)	5293 (76.20)	1.00	
CG + GG	1820 (21.47)	1653 (23.80)	0.87 (0.81–0.94)	0.001
*FMN1* rs117648907				
CC	8030 (94.73)	6658 (95.85)	1.00	—
CT	440 (5.19)	285 (4.10)	1.33 (1.14–1.55)	<0.001
TT	7 (0.08)	3 (0.04)	2.02 (0.52–7.83)	0.309
Trend test				<0.001
Dominant model				
CC	8030 (94.73)	6658 (95.85)	1.00	—
CT + TT	447 (5.27)	288 (4.15)	1.33 (1.15–1.55)	<0.001
*MGAT3* rs34943118				
TT	4571 (53.95)	3943 (56.84)	1.00	—
TC	3273 (38.63)	2571 (37.06)	1.08 (1.01–1.16)	0.019
CC	629 (7.42)	423 (6.10)	1.25 (1.10–1.43)	0.001
Trend test				<0.001
Dominant model				
TT	4571 (53.95)	3943 (56.84)	1.00	—
TC + CC	3902 (46.05)	2994 (43.16)	1.11 (1.04–1.18)	0.002
NUG^b^				
0	1600 (18.88)	1485 (21.41)	1.00	—
1	6050 (71.40)	4919 (70.91)	1.14 (1.05–1.24)	0.001
2	790 (9.32)	521 (7.51)	1.41 (1.24–1.61)	<0.001
3	33 (0.39)	12 (0.17)	2.70 (1.39–5.25)	0.004
Trend test				<0.001
0–1	7650 (90.29)	6404 (92.32)	1.00	—
2–3	823 (9.71)	533 (7.68)	1.30 (1.16–1.46)	<0.001

Abbreviations: CI, confidence interval; NUG, number of unfavorable genotypes; OR, odds ratio; PanC, pancreatic cancer; SNP, single‐nucleotide polymorphism.

^a^ Adjusted by age, sex, study, and the top five significant principal components.

^b^ Unfavorable genotypes were rs211304 CC, rs117648907 CT + TT, and rs34943118 CC.

Furthermore, to identify association between NUG and PanC risk, we conducted stratified analyses by age (<60, 60–70, and >70 years) and sex. The risk associated with the NUG was more evident in age subgroups of 60–70 years (OR = 1.33, 95% CI = 1.11–1.61, *p* = 0.003) and >70 years (OR = 1.47, 95% CI = 1.21–1.79, *p* < 0.001) than in <60 years (OR = 1.08, 95% CI = 0.87–1.34, *p* = 0.508), and the interaction between age group and genotypes was statistically significant (*P*_inter_ = 0.034, Table S8). However, no interaction was found between sex and genotypes (*P*_inter_ = 0.230, Table S8).

### 3.3 eQTL and functional analyses

**Figure 3:**
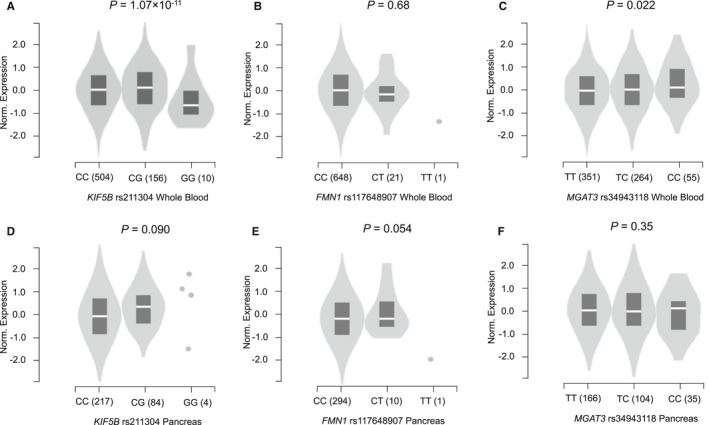
eQTL results of these three novel independent SNPs from GTEx Project. eQTL results in the whole blood cells (*n* = 670): (a)*KIF5B*rs211304 (*p* = 1.07 × 10^−11^,*β* = 0.11), (b)*FMN1*rs117648907 (*p* = 0.68,*β* = −0.044), and (c)*MGAT3*rs34943118 (*p* = 0.022,*β* = 0.085); eQTL results in pancreas (*n* = 305): (d)*KIF5B*rs211304 (*p* = 0.090,*β* = 0.063), (e)*FMN1*rs117648907 (*p* = 0.054,*β* = 0.31), and (f)*MGAT3*rs34943118 (*p* = 0.35,*β* = −0.067). eQTL, expression quantitative trait loci; GTEx, Genotype‐Tissue Expression; SNP, single‐nucleotide polymorphism.

We used eQTL analysis to further evaluate potential functions of the three identified SNPs. In the 1000 Genomes Project, the rs211304 G allele was correlated with higher *KIF5B* mRNA expression levels in lymphoblastoid cells (*n* = 373, *p* = 0.009; Figure S5A), but no such correlation was evident for the *FMN1* rs117648907 T or the *MGAT3* rs34943118 C allele (Figures S5B, C). In the GTEx Project, the eQTL results in whole blood cells revealed that the *KIF5B* rs211304 G and *MGAT3* rs34943118 C alleles, but not the *FMN1* rs117648907 T allele, were correlated with higher mRNA expression levels of their corresponding genes (*n* = 670, *p* = 1.07 × 10^−11^ and 0.022; Figure 3a–c). However, none of the three SNPs was significantly correlated with the mRNA expression level of its corresponding gene in normal pancreas samples (Figure 3d–f), although the rs211304 G allele was associated with higher *KIF5B* expression in both normal liver and colon tissues (*p* < 0.050; Figures S5D–E). In addition, we performed the colocalization analysis for the eQTL signal in the 670 whole blood samples from the GTEx Project and GWAS signal in the PanC risk association. The results indicated that there was a strong evidence of the eQTL‐association pair on the locus on Chromosome 10 (posterior probability = 81%), but not on Chromosome 15 (posterior probability = 0.115%) or on Chromosome 22 (posterior probability = 1.73%) (Table S9).

Furthermore, we assessed function prediction for the three novel SNPs and other SNPs in high LD (*r* ^2^ > 0.80) with them by using online bioinformatics tools. As a result, the *KIF5B* rs211304 and the *MGAT3* rs34943118 may affect enhancer histone marks, because they are located at DNase hypersensitive sites, and cause changes in binding motifs, such as those for HNF4A and ATF2 (Table S10; Figure S6). In addition, *FMN1* rs117648907 may also affect enhancer histone marks (Table S10). The data from the ENCODE Project showed that the three SNPs are located in regions enriched for active histone marks, including histone H3 monomethylated at K4 (H3K4me1; Figure S7).

## 4 DISCUSSION

In the current study, we identified three novel PanC risk‐associated SNPs (i.e., *KIF5B* rs211304 C > G, *FMN1* rs117648907 C > T, and *MGAT3* rs34943118 T > C) in cadherin‐related genes by utilizing genotyping data from publically available PanScan and PanC4 datasets. We also found that *KIF5B* rs211304 G allele and *MGAT3* rs34943118 C allele were associated with higher expression levels of their corresponding genes in whole blood cells, which provides biological plausibility for the observed associations.

*KIF5B* located at 10p11.22 encodes the KIF5B protein, one of the kinesin1 family members, that plays important roles in the mitochondria and lysosome membrane transport.^46^, ^47^ In the current study, the G allele of the rs211304 located in the 2‐kb upstream region of *KIF5B* was found to be correlated with a decreased PanC risk, possibly by increasing *KIF5B* gene expression. Based on the ENCODE Project data, rs211304 is likely located in the region enriched for H3K4Me1 to enhance transcriptional activity. In addition, *KIF5B* rs211304 is predicted to alter the binding capacity of the DNA promoter region for the HNF4A motif, indicating a potential role of this SNP in regulating *KIF5B* expression. One study has reported that the *KIF5B* gene mediates plasma membrane translocation of the glucose transporter type 4, ablation of which may lead to glucose intolerance, insulin resistance, and diabetes.^48^ Another study demonstrated that the deletion of *Kif5b*‐induced obesity and insulin resistance.^49^ These findings indicate that genetic variants in *KIF5B* could be candidate biomarkers for PanC risk but need to be validated in additional functional studies.

*FMN1* located at 15q13.3 encodes the formin1 protein that plays roles in the polymerization of linear actin filaments and the formation of adherens junctions.^50^ In the current study, the T allele of the rs117648907 located in the intron of *FMN1* was found to be correlated with an increased PanC risk. Previously published studies have found that chromosome 15q13.3 contains multiple colorectal cancer risk loci (*SCG5* rs4779584 and *GREM1* rs10318)^51^, ^52^ and that *FMN1* rs2306277 possibly participated in the formation of predisposition to prostate cancer.^53^ Although these variants in the same loci are located in distance from and not in LD with the *FMN1* rs117648907 SNP in our current study, the 15q13.3 region may harbor potential susceptibility loci involved in multiple cancers. More importantly, *FMN1* has also been identified as a candidate gene for susceptibility to chronic pancreatitis ^54^ predisposing the hosts to 13.3‐fold increased risk of PanC.^55^ However, no significant correlation was found between the rs117648907 minor T allele and *FMN1* mRNA expression levels in either the 1000 Genomes or GTEx Projects, although rs117648907 is predicted to affect on enhancer histone marks. Therefore, additional mechanistic studies are warranted to validate this finding.

*MGAT3* located at 22q13.1 encodes the MGAT3 protein, also called glycosyltransferase GnT‐III that can catalyze the addition of the bisecting GlcNAc to N‐glycans to suppress further branching, thus, leading tumorigenesis.^56^ In the current study, the C allele of rs34943118 located in an intron of *MGAT3* was found to be correlated with an increased risk of PanC and increased mRNA expression of *MGAT3*. Aberrant expression of *MGAT3* was reported to mediate the development of many other cancers.^57^, ^58^ Two *MGAT3* variants that are in moderate LD (rs34692520, *r* ^2^ = 0.56 and rs12484278, *r* ^2^ = 0.41) with rs34943118 have been identified to be associated with Immunoglobulin G glycosylation patterns^59^ that have important regulatory functions in cancers and autoimmune diseases, including PanC and pancreatitis.^60^, ^61^ Furthermore, we found that the change from rs34943118 T to C might alter the binding motif for ATF2, but the biological plausibility of the observed association between the C allele of rs34943118 and PanC risk needed to be further validated.

The current study has some limitations. First, only the variables of age and sex were available for the analysis, and other critical clinical variables could not be further analyzed such as family history of cancer, diabetes history, dietary factors, and smoking status. Second, the two available GWAS datasets used were generated from European ancestry populations, and thus, our findings may not be generalized to other ethnic populations. Third, the ability of the publicly available data to accurately predict the biological functions of the SNPs is limited. Finally, none of SNPs could surpass the more stringent Bonferroni or FDR corrections. Although the BFDP approach was used for multiple testing corrections and eQTL functional evidence was also provided, additional mechanistic studies are warranted to validate our findings.

## PanScan

The PanScan study was funded in whole or in part with federal funds from the National Cancer Institute (NCI) and National Institutes of Health (NIH), contract number HHSN261200800001E. This study was also supported by NIH/NCI K07CA140790, the American Society of Clinical Oncology Conquer Cancer Foundation, the Howard Hughes Medical Institute, the Lustgarten Foundation, the Robert T. and Judith B. Hale Fund for Pancreatic Cancer Research and Promises for Purple. A full list of acknowledgments for each participating study is provided in the Supplementary Note of the manuscript with PubMed ID: 25086665. The dbGaP accession number for this study is phs000206.v5.p3.

## PanC4

The cases and controls for the PanC4 study were drawn from the following studies: Johns Hopkins National Familial Pancreas Tumor Registry, Mayo Clinic Biospecimen Resource for Pancreas Research, Ontario Pancreas Cancer Study (OPCS), Yale University, MD Anderson Case Control Study, Queensland Pancreatic Cancer Study, University of California San Francisco Molecular Epidemiology of Pancreatic Cancer Study, International Agency of Cancer Research and Memorial Sloan Kettering Cancer Center. The PanC4 study was supported by NCI R01CA154823. Genotyping services were provided by the Center for Inherited Disease Research (CIDR). CIDR was funded by a federal contract from the NIH to The Johns Hopkins University, contract number HHSN2682011000111. The dbGaP accession number for this study is phs000648.v1.p1.

## TCGA

The results published here are in whole or part based on data generated by TCGA project established by the NCI and the National Human Genome Research Institute (NHGRI). Information about TCGA and the investigators and institutions that constitute TCGA Research Network can be found at “http://cancergenome.nih.gov.” The TCGA SNP data analyzed here are requested through dbGaP, accession number phs000178.v1.p1.

## AUTHOR CONTRIBUTIONS

LZ mainly performed the data analysis process and wrote the manuscript. QW and WL supervised the work and wrote the manuscript. HL, SL, PM, and KW co‐supervised the work and edited the manuscript. All authors edited and contributed to the final version of the manuscript.

## Supporting information

Supplementary Material

## Data Availability

The PanScan and the PanC4 datasets are available through the database of Genotypes and Phenotypes (dbGaP; accession #: phs000206.v5.p3 and phs000648.v1.p1, respectively). Approval for the current study protocol was obtained from the Duke University Health System Institutional Review Board (Pro00054575).
