# Supplementary material for: Associations between genetic variants of KIF5B, FMN1, and MGAT3 in the cadherin pathway and pancreatic cancer risk
Source: Cancer Med. 2020 Nov 16;9(24):9620–31. doi: 10.1002/cam4.3603 (PMC7774717; doi:10.1002/cam4.3603)
Supplement: Supplementary file 1 — Supplementary Material [file CAM4-9-9620-s001.docx]

| **Table S1**. Distributions of demographic characteristics among the three pancreatic cancer genotyping datasets | | | | | | | | | | | |
| --- | --- | --- | --- | --- | --- | --- | --- | --- | --- | --- | --- |
| **Characteristic** | **PanScan I** | | |  | **PanScan II/III** | | |  | **PanC4 ^a^** | | |
|  | **Case (%)** | **Control (%)** | ***P* ^b^** |  | **Case (%)** | **Control (%)** | ***P* ^b^** |  | **Case (%)** | **Control (%)** | ***P* ^b^** |
| **Age** |  |  | 0.009 |  |  |  | <.0001 |  |  |  | <.0001 |
| **< 60** | 330 (18.8) | 265 (14.9) |  |  | 820 (27.3) | 564 (33.9) |  |  | 1134 (30.5) | 1260 (35.9) |  |
| **60-70** | 678 (38.5) | 715 (40.2) |  |  | 973 (32.5) | 548 (32.9) |  |  | 1344 (36.1) | 1170 (33.4) |  |
| **> 70** | 752 (42.7) | 800 (44.9) |  |  | 1202 (40.1) | 554 (33.2) |  |  | 1241 (33.4) | 1068 (30.6) |  |
| **Sex** |  |  | 0.719 |  |  |  | 0.229 |  |  |  | 0.110 |
| **Male** | 899 (51.1) | 921 (51.7) |  |  | 1549 (51.7) | 893 (53.6) |  |  | 2144 (57.6) | 1953 (55.7) |  |
| **Female** | 861 (48.9) | 859 (48.3) |  |  | 1446 (48.3) | 773 (46.4) |  |  | 1578 (42.4) | 1547 (44.3) |  |
| **Total** | **1760** | **1780** |  |  | **2995** | **1666** |  |  | **3722** | **3500** |  |
| ^a^ Five missing values (three in cases and two in controls) of age in the PanC4 dataset. | | | | | | | | | | | |
| ^b^ Chi-square test. | | | | | | | | | | | |

| **Table S2.** List of 109 selected genes in the cadherin pathway | | | |
| --- | --- | --- | --- |
| **Dataset** | **Name of the pathway** | **Gene Number** | **Selected genes**^a^ |
| **MSigDB**^b^ |  |  | *ABI1, ACTN1, AJUBA, AKT1, AKT2, ANXA1, ANXA2, AP1M1, AQP3, AQP5, ARF6, AXIN1, BAIAP2, BAIAP2L1, CALM1, CALM2, CALM3, CAMK2G, CAMSAP3, CASR, CCND1, CDC42, CDC42EP1, CDH1, CDH2, CNN3, CNR1, CRK, CTNNA1, CTNNB1, CTNND1, CTTN, CYFIP2, DAGLA, DAGLB, DCTN1, DIAPH1, DLG1, EFNA1, EGF, EGFR, ENAH, EPCAM, EPHA2, EXOC3, EXOC4, FER, FGFR1, FMN1, FYN, GAP43, GIT1, GJA1, GRIA2, GSN, HGF, IGF1R, IQGAP1, ITGAE, ITGB7, JUP, KIF5B, KIFC3, KLHL20, KRT18, LIMA1, LPP, LRP5, MAPK8, MAPRE1, MET, MGAT3, MYL2, MYO6, NCK1, NCKAP1, NME1, PAK4, PDLIM1, PDLIM5, PIK3CA, PIK3R1, PIP5K1A, PIP5K1C, PKP3, PLCG1, PLEKHA7, PPP1CA, PTPN1, PVRL2* ^d^*, RAB10, RAC1, RAPGEF1, RHOA, ROCK1, S100A11, SRC, STX4, STXBP6, TACSTD2, TIAM1, TJP1, TMOD3,TRIM29, VASP, VAV2,VCL, WASF2, ZYX* |
| GO | GO_CADHERIN_BINDING_INVOLVED_IN_CELL_CELL_CELL_ADHESION | 19 |  |
| BIOCARTA | NO DATA | 0 |  |
| PID | NO DATA | 0 |  |
| REACTOME | NO DATA | 0 |  |
| KEGG | NO DATA | 0 |  |
| **GeneCards**^c^ |  |  |  |
| PathCards | N-cadherin signaling events | 33 |  |
| PathCards | E-cadherin signaling in keratinocytes | 19 |  |
| PathCards | E-cadherin signaling in the nascent adherens junction | 33 |  |
| PathCards | Stabilization and expansion of the E-cadherin adherens junction | 40 |  |
| **Total**^a^ |  | 109 |  |
| Abbreviations: MSigDB, The Molecular Signatures Database.  ^a^ After removing 44 duplicated genes.  ^b^ http://software.broadinstitute.org/gsea/msigdb/search.jsp (v7.0).  ^c^ https://www.genecards.org/.  ^d^ *PVRL2,* also known as *NECTIN2.*  Keyword: cadherin.  Organism: Homo sapiens. | | | |

| **Table S3.** Associations between 20 principal components in three genotyping datasets and PanC risk | | | | |
| --- | --- | --- | --- | --- |
| **PC**^a^ | **Parameter Estimate** | **Standard Error** | **Chi-Square** | ***P*** |
| PC1 | 9.58 | 19.15 | 0.25 | 0.620 |
| PC2 | -4.63 | 17.18 | 0.07 | 0.790 |
| PC3 | -15.56 | 2.95 | 27.86 | <0.0001 |
| PC4 | -0.42 | 2.89 | 0.02 | 0.880 |
| PC5 | 4.09 | 5.47 | 0.56 | 0.460 |
| PC6 | -7.43 | 2.15 | 11.99 | 0.0001 |
| PC7 | -8.14 | 2.26 | 12.95 | 0.0001 |
| PC8 | 1.76 | 4.70 | 0.14 | 0.710 |
| PC9 | 3.47 | 2.07 | 2.82 | 0.090 |
| PC10 | 27.46 | 6.09 | 20.36 | <0.0001 |
| PC11 | 4.80 | 2.17 | 4.90 | 0.030 |
| PC12 | 34.67 | 5.58 | 38.57 | <0.0001 |
| PC13 | 3.36 | 3.82 | 0.77 | 0.380 |
| PC14 | -6.64 | 3.32 | 4.00 | 0.050 |
| PC15 | 1.89 | 2.98 | 0.40 | 0.530 |
| PC16 | -4.58 | 3.02 | 2.31 | 0.130 |
| PC17 | 0.81 | 2.63 | 0.09 | 0.760 |
| PC18 | 8.92 | 3.57 | 6.23 | 0.010 |
| PC19 | 1.88 | 2.82 | 0.45 | 0.500 |
| PC20 | -5.56 | 2.61 | 4.54 | 0.030 |
| Abbreviations: PanC, pancreatic cancer; PC, principal components.  **^a^** PC3, PC6, PC7, PC10 and PC12 were selected as the top five PC for the adjustment in the multivariate analysis. | | | | |

| **Table S4. Independent SNPs identified in the current study in the model in the presence of other SNPs identified previous studies** | | | | | |  |
| --- | --- | --- | --- | --- | --- | --- |
| **SNP** | | **Gene** | **Location** | **Category ^a^** | **OR (95% CI)** ^b^ | ***P*** ^b^ |
| rs5757573 **^c^** | | *PDGFB* | 22q13.1 | T/C | 1.07 (1.01-1.12) | 0.019 |
| rs6001516 **^c^** | | *PDGFB* | 22q13.1 | C/T | 1.15 (1.04-1.26) | 0.005 |
| rs79447092 **^c^** | | *ARHGEF7* | 13q34 | T/A | 0.74 (0.64-0.85) | <.001 |
| rs9895829 **^c^** | | *TP53* | 17p13.1 | A/G | 0.83 (0.75-0.93) | 0.001 |
| rs3818626 **^c^** | | *SMC2* | 9q31.1 | T/C | 1.13 (1.08-1.19) | <.001 |
| rs3124761 **^c^** | | *SLC2A6* | 9q34.2 | C/T | 1.15 (1.08-1.23) | <.001 |
| rs17458086 **^c^** | | *SLC2A13* | 12q12 | T/C | 1.32 (1.10-1.59) | 0.003 |
| rs1630747 **^c^** | | *SLC5A3* | 21q22.11 | A/C | 0.92 (0.87-0.97) | 0.003 |
| rs35075084 **^c^** | | *MAP2* | 2q34 | T/- | 0.77 (0.66-0.92) | 0.003 |
| rs2727572 **^c^** | | *PRKAG2* | 7q36.1 | C/T | 1.07 (1.02-1.12) | 0.005 |
| rs34852782 **^c^** | | *PRKAG2* | 7q36.1 | A/- | 1.10 (1.04-1.16) | <.001 |
| rs62068300 **^c^** | | *RPTOR* | 17q25.3 | G/A | 0.93 (0.88-0.97) | 0.003 |
| rs3751936 **^c^** | | *RPTOR* | 17q25.3 | G/C | 0.92 (0.87-0.97) | 0.002 |
| **rs211304 ^d^** | | ***KIF5B*** | **10p11.22** | **C/G** | **0.90 (0.84-0.97)** | **0.004** |
| **rs117648907 ^d^** | | ***FMN1*** | **15q13.3** | **C/T** | **1.30 (1.11-1.52)** | **0.001** |
| **rs34943118 ^d^** | | ***MGAT3*** | **22q13.1** | **T/C** | **1.09 (1.04-1.16)** | **0.001** |
| Abbreviations: SNP, single nucleotide polymorphism; OR, odds ratio; CI, confidence interval. ^a^ Reference allele/effect allele.  ^b^ Stepwise analysis adjusted by age, sex, study and the top five principal components (PC3, PC6, PC7, PC10 and PC12 shown in Table S3) and 13 SNPs (rs5757573, rs6001516, rs79447092, rs9895829, rs3818626, rs3124761, rs17458086, rs1630747, rs35075084, rs2727572, rs34852782, rs62068300 and rs3751936) previously reported in the same model (PMID: 29168174, 30794721, 30972876 and 30997723, respectively). ^c^ SNPs previously reported in the same model (PMID: 29168174, 30794721, 30972876 and 30997723, respectively). ^d^ Three novel independent SNPs identified in the current study. | | | | | | |

| **Table S5. Independent SNPs identified in the current study with COJO method in the model in the presence of other SNPs identified previous studies** | | | | | |
| --- | --- | --- | --- | --- | --- |
| **Chr** | **SNP** | **Position** | **Frequency** | ***P*** | **LD_r** |
| 2 | rs35075084 ^a^ | 210473015 | 0.02 | 0.001 | 0 |
| 7 | rs2727572 ^a^ | 151298246 | 0.46 | 0.001 | 0 |
| 7 | rs34852782 ^a^ | 151566171 | 0.29 | 2.04x10^-4^ | 0 |
| 9 | rs3818626 ^a^ | 106856633 | 0.45 | 2.24x10^-6^ | 0 |
| 9 | rs3124761 ^a^ | 136339755 | 0.16 | 5.17x10^-7^ | 0 |
| 12 | rs17458086 ^a^ | 40428639 | 0.02 | 5.63x10^-4^ | 0 |
| 13 | rs79447092 ^a^ | 111809308 | 0.03 | 1.47x10^-4^ | 0 |
| 17 | rs9895829 ^a^ | 7578679 | 0.05 | 1.37x10^-4^ | 0 |
| 17 | rs62068300 ^a^ | 78574727 | 0.31 | 7.51x10^-4^ | -0.01 |
| 17 | rs3751936 ^a^ | 78938204 | 0.25 | 6.99x10^-4^ | 0 |
| 21 | rs1630747 ^a^ | 35457991 | 0.26 | 5.53x10^-4^ | 0 |
| 22 | rs5757573 ^a^ | 39633622 | 0.38 | 0.006 | 0.37 |
| 22 | rs6001516 ^a^ | 39644203 | 0.08 | 0.009 | 0.11 |
| **10** | **rs211304** | **32347322** | **0.12** | **6.94x10^-4^** | **0** |
| **15** | **rs117648907** | **33277710** | **0.02** | **2.11x10^-4^** | **0** |
| **22** | **rs34943118** | **39878998** | **0.26** | **4.47x10^-4^** | **0** |
| Abbreviations: COJO, conditional and joint; Chr, chromosome; SNP, single nucleotide polymorphism, LD, linkage disequilibrium.  ^a^ SNPs previously reported in the same model (PMID: 29168174, 30794721, 30972876 and 30997723, respectively). | | | | | |

| **Table S6. Significant results in the gene-based test with VEGAS method** | | | | | | | | | | | |
| --- | --- | --- | --- | --- | --- | --- | --- | --- | --- | --- | --- |
| Chr | Gene | nSNPs | nSims | Start | Stop | Test^a^ | Gene-*P* value | Best-SNP | SNP-*P* value^b^ | SNP*-P* value^c^ | BFDP^d^ |
| 3 | *RHOA* | 84 | 1.00x10^5^ | 49394578 | 49451526 | 480.01 | 2.67x10^-3^ | rs35169793 | 5.97x10^-4^ | 5.97x10^-4^ | 0.95 |
| **22** | ***MGAT3*** | **65** | **1.00x10^5^** | **39851324** | **39890199** | **224.98** | **3.86x10^-3^** | **rs2017711** | **5.27x10^-5^** | **5.27x10^-5^** | **0.69** |
| 1 | *WASF2* | 140 | 1.00x10^5^ | 27728733 | 27818678 | 716.25 | 5.48x10^-3^ | 1:27735922 | 6.11x10^-3^ | 6.11x10^-4^ | 0.91 |
| 20 | *MAPRE1* | 99 | 1.00x10^5^ | 31405698 | 31440211 | 286.88 | 7.79x10^-3^ | rs4911268 | 4.08x10^-3^ | 0.101 | 1.00 |
| **10** | ***KIF5B*** | **130** | **1.00x10^5^** | **32295937** | **32347371** | **434.22** | **1.43x10^-2^** | **rs211304** | **6.93x10^-4^** | **6.93x10^-4^** | **0.79** |
| 9 | *RAPGEF1* | 445 | 1.00x10^5^ | 134450156 | 134614925 | 1016.37 | 2.75x10^-2^ | rs10655612 | 1.89x10^-3^ | 1.89x10^-3^ | 0.98 |
| 12 | *AQP5* | 8 | 1.00x10^5^ | 50353278 | 50361465 | 25.05 | 3.15x10^-2^ | rs923911 | 1.76x10^-3^ | 1.76x10^-3^ | 0.96 |
| **15** | ***FMN1*** | **2062** | **1.00x10^5^** | **33055744** | **33488934** | **3147.47** | **3.40x10^-2^** | **rs117648907** | **2.10x10^-4^** | **2.11x10^-4^** | **0.63** |
| 17 | *GIT1* | 23 | 1.00x10^5^ | 27898486 | 27918610 | 53.72 | 4.18x10^-2^ | rs3744626 | 1.64x10^-2^ | 0.184 | 1.00 |
| Abbreviations: VEGAS, versatile gene-based association study; Chr, chromosome; SNP, single nucleotide polymorphism; nSims, number of simulations; BFDP, Bayesian false-discovery probability. ^a^ Gene-based test statistic;  ^b^ SNP-*P* value from Gene-based test;  ^c^ SNP-*P* value from meta-analysis of three GWAS datasets;  ^d^ BFDP approach with a prior probability of 0.01 and an upper bound of 3.0 for multiple correction testing from meta-analysis. | | | | | | | | | | | |

| **Table S7. Associations between combined three novel SNPs identified in the current study with 13 SNPs previously discovered and PanC risk in the same model** | | | | | | |  |
| --- | --- | --- | --- | --- | --- | --- | --- |
| **NUG** ^a^ | **Case (%)** | **Control (%)** | **Univariate analysis** | | **Multivariate analysis ^b^** | | |
|  |  |  | **OR (95% CI)** | ***P*** | **OR (95% CI)** | ***P*** | |
| 2-6 | 1167 (13.8) | 1227 (17.6) | 1 | - | 1 | - | |
| 7 | 1247 (14.7) | 1157 (16.6) | 1.13 (1.01-1.27) | 0.030 | 1.13 (1.01-1.27) | 0.033 | |
| 8 | 1899 (22.4) | 1646 (23.6) | 1.21 (1.09-1.35) | <0.001 | 1.22 (1.10-1.36) | <0.001 | |
| 9 | 1983 (23.4) | 1522 (21.8) | 1.37 (1.23-1.52) | <0.001 | 1.38 (1.25-1.54) | <0.001 | |
| 10 | 1360 (16.0) | 935 (13.4) | 1.53 (1.36-1.72) | <0.001 | 1.55 (1.38-1.74) | <0.001 | |
| 11 | 625 (7.4) | 382 (5.5) | 1.72 (1.48-2.00) | <0.001 | 1.73 (1.49-2.02) | <0.001 | |
| 12-14 | 202 (2.4) | 101 (1.4) | 2.10 (1.63-2.71) | <0.001 | 2.16 (1.68-2.78) | <0.001 | |
| Trend test |  |  |  | <0.001 |  | <0.001 | |
| 2-10 | 7656 (90.2) | 6487 (93.1) | 1 | - | 1 | - | |
| 11-14 | 827 (9.8) | 483 (6.9) | 1.45 (1.29-1.63) | <0.001 | 1.46 (1.29-1.64) | <0.001 | |
| Abbreviations: SNP, single nucleotide polymorphism**;** PanC, pancreatic cancer; NUG, number of unfavorable genotype; OR, odds ratio; CI, confidence interval. ^a^ Risk genotypes were rs35075084 TT, rs2727572 CT+TT, rs34852782 A-+--, rs9895829 AA, rs62068300 GG, rs3751936 GG, rs3124761 CT+TT, rs17458086 TC+CC, rs1630747 AA, rs5757573 TC+CC, rs6001516 TC+TT, rs3818626 TC+CC, rs79447092 TT, rs211304 CC, rs117648907 CT+TT and rs34943118 CC. ^b^ Adjusted for age, sex, study and the top five principal components. | | | | | | | |

| **Table S8.** Stratified analysis for associations between NUG and PanC risk by age and sex | | | | | | | | | |
| --- | --- | --- | --- | --- | --- | --- | --- | --- | --- |
| **Characteristic** | **NUG 0-1** | | **NUG 2-3** | | **Univariate analysis** | | **Multivariate analysis^a^** | | ***P*_inter_^b^** |
|  | **Case (%)** | **Control (%)** | **Case (%)** | **Control (%)** | **OR (95% CI)** | ***P*** | **OR (95% CI)** | ***P*** |  |
| **Age (years)** |  |  |  |  |  |  |  |  | 0.034 |
| **<60** | 2088 (27.3) | 1920 (30.0) | 194 (23.6) | 168 (31.5) | 1.06 (0.86-1.32) | 0.586 | 1.08 (0.87-1.34) | 0.508 |  |
| **60-70** | 2674 (35.0) | 2230 (34.8) | 321 (39.0) | 200 (37.5) | 1.34 (1.11-1.61) | **0.002** | 1.33 (1.11-1.61) | **0.003** |  |
| **>70** | 2885 (37.7) | 2252 (35.2) | 308 (37.4) | 165 (31.0) | 1.46 (1.20-1.77) | **<.001** | 1.47 (1.21-1.79) | **<.001** |  |
| **Sex** |  |  |  |  |  |  |  |  | 0.230 |
| **Male** | 4145 (54.3) | 3496 (54.6) | 438 (53.2) | 265 (49.7) | 1.39 (1.19-1.63) | **<.001** | 1.38 (1.18-1.62) | **<.001** |  |
| **Female** | 3499 (45.7) | 2908 (45.4) | 385 (46.8) | 268 (50.3) | 1.19 (1.01-1.41) | **0.034** | 1.21 (1.03-1.43) | **0.023** |  |
| Abbreviations: NUG: number of unfavorable genotypes; PanC, pancreatic cancer; OR, odds ratio; CI, confidence interval. | | | | | | | | | |
| ^a^ Adjusted for age, sex, study and the top five principal components. | | | | | | | | | |
| ^b^ *P*_inter_: *P* value for interaction analysis between characteristics and NUGs. | | | | | | | | | |

| **Table S9.** Colocalization results of the eQTL^a^ and GWAS signals in the current study | | | | | | |
| --- | --- | --- | --- | --- | --- | --- |
| **Chr** | **Gene** | **PP0 (%)** | **PP1 (%)** | **PP2 (%)** | **PP3 (%)** | **PP4 (%)** |
| 15 | *FMN1* | 98.4 | 1.43 | 0.00794 | 0 | 0.115 |
| 22 | *MGAT3* | 91.9 | 6.32 | 0.0532 | 0.00193 | 1.73 |
| **10** | ***KIF5B*** | **0.00273** | **0.0000958** | **18.4** | **0.565** | **81** |
| Abbreviations: eQTL, expression quantitative trait loci; GWAS, genome-wide association study; Chr, chromosome; PP, posterior probability. ^a^ eQTL data from the 670 whole blood samples in the GTEx Project. | | | | | | |

| **Table S10.** Functional prediction of the three independent SNPs in the present study and other SNPs in high LD (r^2^ > 0.80) with these three SNPs | | | | | | | | | | | |
| --- | --- | --- | --- | --- | --- | --- | --- | --- | --- | --- | --- |
| **SNP** | **Chr** | **Gene** | **SNP-info^a^** | **Regulme-DB score^b^** | **HaploReg v4.1^c^** | | | | | | |
|  |  |  |  |  | **LD (r^2^)** | **Promoter histone marks** | **Enhancer histone marks** | **DNase** | **Proteins bound** | **Motifs changed** | **Selected eQTL hits** |
| **rs211304** | **10** | ***KIF5B*** | **TFBS** | **6** | **1.00** |  | **ESDR, GI, LIV, BLD** | **BLD** |  | **AP-4, LBP-1, SMC3, TCF12** | **1 hit** |
| rs211268 | 10 | *KIF5B* |  | 7 | 0.97 |  |  |  |  | Zfp691 | 1 hit |
| rs211255 | 10 | *KIF5B* |  | 3a | 0.97 |  | ESC, ESDR, IPSC, BRN, SKIN, MUS | SKIN |  | CHX10, Nanog, Pou2f2, Pou3f1, Sox, TCF4 | 1 hit |
| rs211385 | 10 | *KIF5B* |  | 3a | 0.98 |  | ESC, ESDR, IPSC, SKIN, FAT | ESC, LNG, IPSC, SKIN, GI |  | AP-1, CTCF, Dbx2, Ets, Gfi1, Hoxb3, Irf, PRDM1, Pax-6, Pou4f3, p300 | 1 hit |
| rs211388 | 10 | *KIF5B* |  | 4 | 0.98 | BRN, GI | ESC, ESDR, IPSC, FAT, BRST, STRM, BRN, SKIN, LIV, GI, ADRL, HRT, KID, MUS, PANC, BLD | ESDR, ESDR, ESC, IPSC, SKIN, ADRL, BRN, HRT, THYM, GI, MUS, MUS, BLD |  | NF-I, Sin3Ak-20 | 1 hit |
| rs211389 | 10 | *KIF5B* |  | 4 | 0.95 |  | ESC, ESDR, IPSC, FAT, BRN, SKIN, HRT, KID, MUS | ESC, ESDR, ESDR, ESC, LNG, IPSC, BLD, BLD, SKIN, SKIN, ADRL, HRT, GI, PLCNT, MUS, LNG, LIV, BRST, VAS, BLD, SKIN, LNG | CTCF, RAD21, SMC3, ELF1, CMYC, MAX |  | 1 hit |
| rs211399 | 10 | *KIF5B* |  | 5 | 1.00 |  | GI |  |  | Egr-1, Ets, PTF1-beta, SIX5 | 1 hit |
| rs211393 | 10 | *KIF5B* |  | 6 | 1.00 |  |  | MUS |  |  |  |
| rs57702189 | 10 | *KIF5B* |  | 7 | 1.00 |  |  |  |  | CHD2 |  |
| rs211362 | 10 | *KIF5B* |  | 7 | 1.00 |  | BLD |  |  | PLZF | 1 hit |
| rs211280 | 10 | *KIF5B* |  | 7 | 1.00 |  |  |  |  | E2F, Ets, Myc, SIRT6 |  |
| rs211284 | 10 | *KIF5B* |  | 4 | 1.00 | BLD | LNG, STRM, BRST, BLD, MUS, SKIN, FAT, GI, HRT, LIV, VAS, BRN, BONE |  |  | GR, Nrf1, SP1, Sin3Ak-20, Znf143 | 1 hit |
| rs200285937 | 10 | *KIF5B* |  | 4 | 0.98 | BLD | BRST, BLD, MUS, FAT, GI, HRT, LIV, VAS, BRN, SKIN, LNG, BONE | SKIN, SKIN |  | Pou3f2 |  |
| rs202023865 | 10 | *KIF5B* |  | 4 | 0.98 | BLD | BRST, BLD, MUS, FAT, GI, HRT, LIV, VAS, BRN, SKIN, LNG, BONE | SKIN, SKIN |  | Hoxa10, Hoxb13, Pou3f2 |  |
| rs211290 | 10 | *KIF5B* |  | 5 | 1.00 |  | BLD, GI, HRT | SKIN |  | LXR, Nkx3 |  |
| rs211293 | 10 | *KIF5B* |  | 4 | 1.00 | IPSC, BLD, VAS, PANC, GI, MUS | ESC, ESDR, LNG, IPSC, FAT, BRST, BLD, STRM, MUS, BRN, SKIN, LIV, GI, ADRL, HRT, KID, PLCNT, CRVX, VAS, BONE | IPSC, HRT |  | Ets, ZBRK1 |  |
| rs211294 | 10 | *KIF5B* |  | 4 | 1.00 | FAT, BRST, GI, BLD, VAS | ESDR, ESC, LNG, IPSC, FAT, STRM, BRST, BLD, MUS, BRN, SKIN, LIV, GI, ADRL, HRT | SKIN |  | PLZF, Pax-4, TEF | 1 hit |
| rs211297 | 10 | *KIF5B* |  | 4 | 1.00 | ESC, ESDR, LNG, IPSC, FAT, STRM, BRST, BLD, MUS, BRN, SKIN, VAS, LIV, GI, ADRL, HRT, KID, PANC, PLCNT, THYM, OVRY, CRVX, BONE |  | ADRL, GI, MUS, MUS, GI, BLD | GATA1 | Ets, Foxj2, Foxp1, Homez, Mef2, Zec |  |
| rs211299 | 10 | *KIF5B* | TFBS | 4 | 1.00 | ESC, ESDR, LNG, IPSC, FAT, STRM, BRST, BLD, MUS, BRN, SKIN, VAS, LIV, GI, ADRL, HRT, KID, PANC, PLCNT, THYM, OVRY, SPLN, CRVX, BONE |  | ESDR,ESDR,ESDR,ESDR,ESC,BRST,BLD,BLD,BLD,BLD,BLD,BLD,BLD,SKIN,SKIN,SKIN,ADRL,BRN,BRN,HRT,GI,GI,KID,LNG,MUS,MUS,PLCNT,GI,THYM,GI,OVRY,MUS,GI,BLD,CRVX,LIV,BRST,MUS,VAS,BLD,BLD | ELF1, PU1, POL2, TCF4, CHD2, POL24H8, TAF1, TBP, HAE2F1 | EWSR1-FLI1, Nrf1, PU.1, RXRA, TATA, WT1, ZNF263, Znf143 |  |
| rs211303 | 10 | *KIF5B* | TFBS | 7 | 1.00 |  | ESDR, GI, LIV, BLD |  |  | Foxa, GR, TCF12 |  |
| rs211308 | 10 | *KIF5B* |  | 6 | 1.00 |  |  |  |  | CACD, NRSF |  |
| rs211309 | 10 | *KIF5B* |  | 4 | 1.00 |  |  | MUS |  |  |  |
| **rs117648907** | **15** | ***FMN1*** |  | **7** | **1.00** |  | **ESDR, LNG, SKIN, GI** |  |  | **Myc** |  |
| rs150962800 | 15 | *FMN1* |  | 5 | 0.88 |  |  |  |  | HNF4 |  |
| **rs34943118** | **22** | ***MGAT3*** |  | **2a** | **1.00** |  | **ESDR, SKIN, FAT, BRN, GI, MUS, OVRY** | **ESDR, LNG, IPSC, SKIN, SKIN, MUS, OVRY, MUS, MUS, VAS, LNG** | **ERALPHA_A** | **AP-4, CTCF, E2F, ERalpha-a, Esr2, Foxa, GR, HNF4, LF-A1, Nanog, RXRA, TAL1** | **3 hits** |
| Abbreviations: SNP, single nucleotide polymorphism; LD, linkage disequilibrium; Chr, chromosome; eQTL, expression quantitative trait loci; TFBS, transcription factor binding sites. | | | | | | | | | | | |
| ^a^ https://snpinfo.niehs.nih.gov/snpinfo/snpfunc.html. | | | | | | | | | | | |
| ^b^ http://regulomedb.org/. | | | | | | | | | | | |
| ^c^ http://archive.broadinstitute.org/mammals/haploreg/haploreg.php. | | | | | | | | | | | |

**Two published PanC GWASs**

**PanC4 data:**

Subjects were from nine case-control

studies;

All studies used the same GWAS chip.

Genotyped SNPs were imputed based on the 1000 Genomes Project by IMPUTE 2.

**PanScan II/III**

2,995 cases/1,666 controls

**PanScan data:**

Three phases: I, II and III;

PanScan III has no controls;

Merged PanScan II and III for PanScan II/III.

Three kinds of GWAS chips.

**PanScan I**

1,760 cases/1,780 controls

**PanC4**

3,722 cases/3,500 controls

Update the Human Genome

Version to GRCh37/hg19

**Figure S1.** Workflow of the data process. Abbreviations: PanC, pancreatic cancer; GWASs, genome-wide association studies; SNP, single nucleotide polymorphism; GRCh37, The Genome Reference Consortium Human Genome Build 37.

**Figure S2.** Distribution plot for imputation Info Quality in the present study.

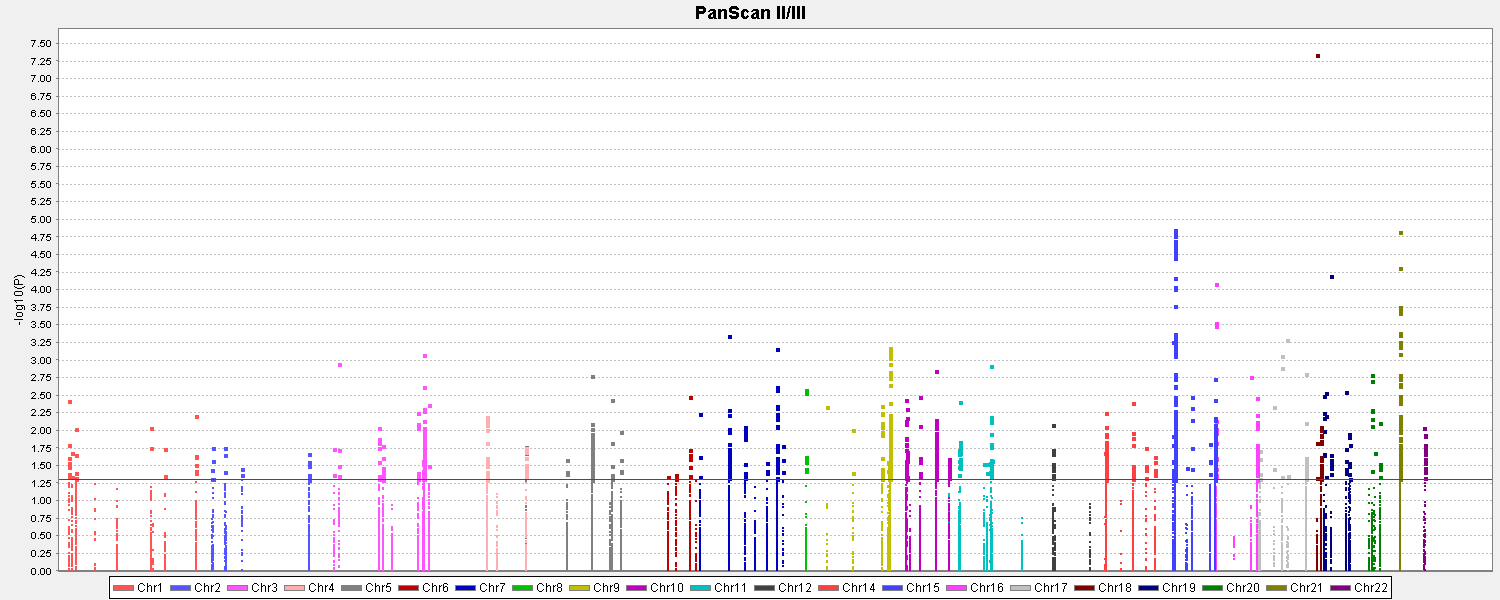

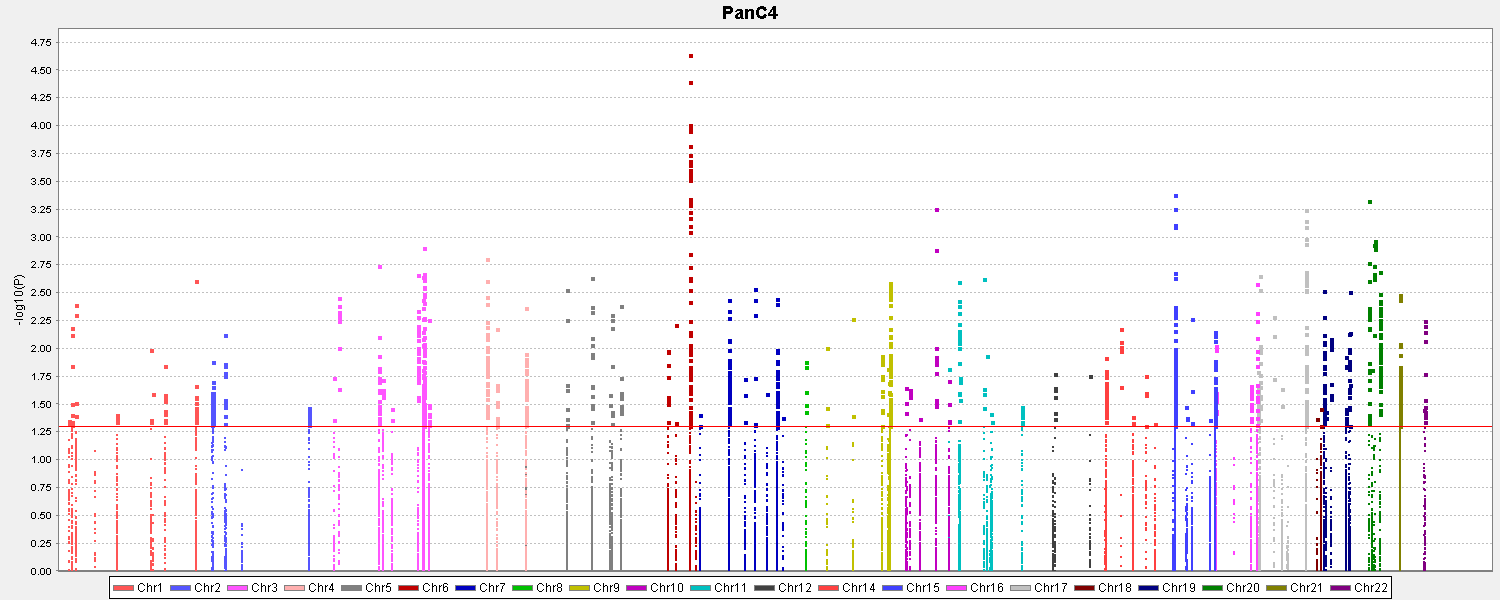

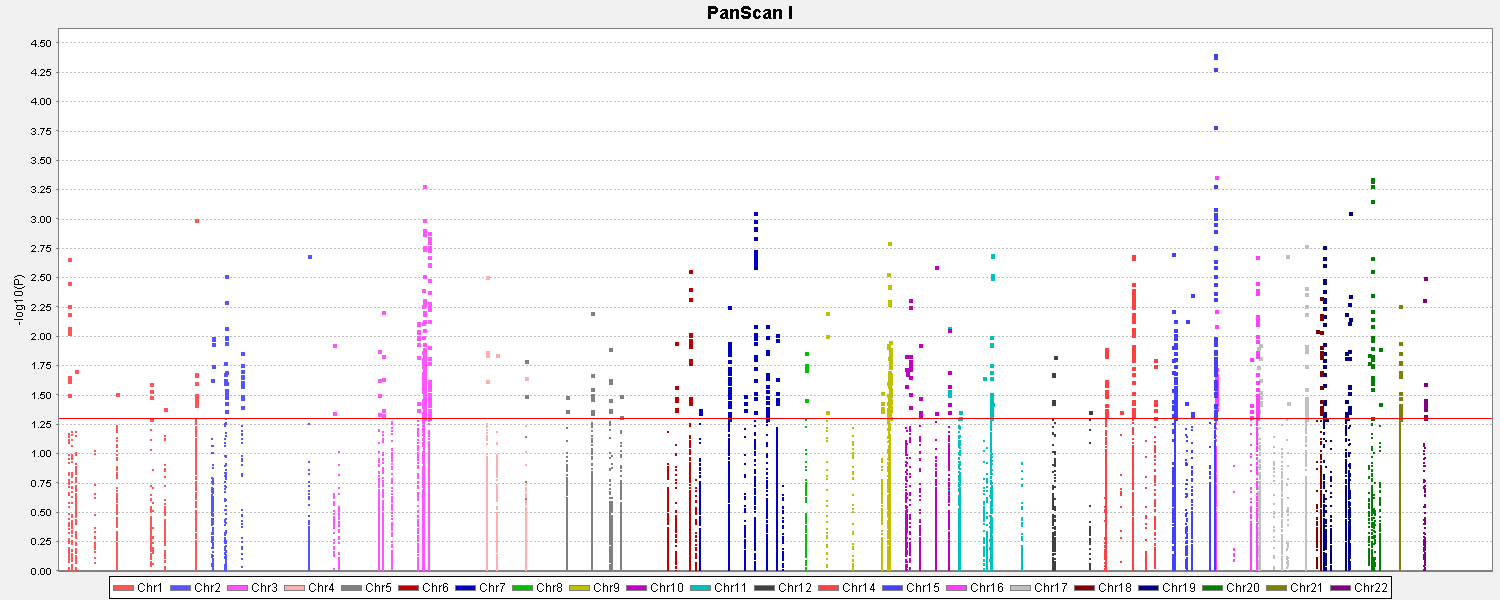


1,362 SNPs with *P* < 0.050

*P* = 0.050

*P* = 0.050

*P* = 0.050

1,884 SNPs with *P* < 0.050

1,642 SNPs with *P* < 0.050

A

C

B

**Figure S3.** Manhattan plot of the association results in the three datasets. The statistical values across the autosomes of associations between SNPs and PanC risk are plotted as −log10 *P* values. The red horizontal line indicates *P* = 0.050. There are (A) 1,362 SNPs with *P* < 0.050 in PanScan I, (B) 1,642 SNPs with *P* < 0.050 in PanScan II/III and (C) 1,884 SNPs with *P* < 0.050 in PanC4. Abbreviations: SNP, single nucleotide polymorphism; PanC, pancreatic cancer.


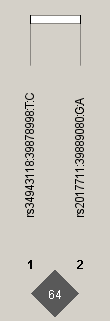

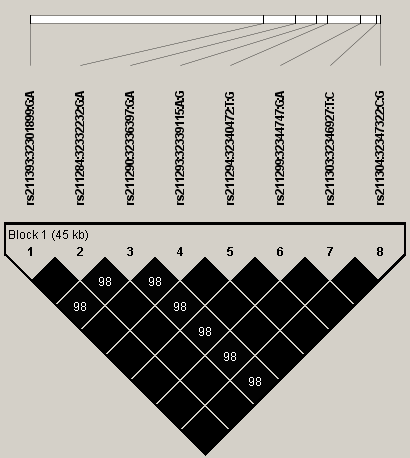


A

B

*KIF5B*

*MGAT3*

**Figure S4.** Linkage disequilibrium (LD) plots between the SNPs in *KIF5B* and *MGAT3* with BFDP < 0.8. (A) rs211304 and (B) rs34943118. Abbreviations: SNP, single nucleotide polymorphism; LD, linkage disequilibrium

**Figure S5.** eQTL results of the three novel independent SNPs. In the 373 transformed lymphoblastoid cells from the 1000 Genomes Project: (A) *KIF5B* rs211304 (n = 373, *P* = 0.009, β = 0.69); (B) *FMN1* rs117648907 (n = 373, *P* = 0.843, β = -0.004, no TT homozygote) and (C) *MGAT3* rs34943118 (n = 373, *P* = 0.118, β = -0.45). In the GTEx Project: *KIF5B* rs211304 (D) (n = 208, *P* = 0.021, β = 0.11, liver) and (E) (n = 368, *P* = 0.019, β = 0.074, colon-transverse). Abbreviations: SNP, single nucleotide polymorphism; GTEx, Genotype-Tissue Expression.

**Figure S6.** Effect analyses of SNPs *KIF5B* rs211304 and *MGAT3* rs34943118 on TF motifs. (A) The change from *KIF5B* rs211304 C allele to G allele may alter the predicted TF-binding motif for HNF4A predicted by using HOCOMOCO-11 collection from the PERFECTOS-APE online tools (https://opera.autosome.ru/perfectosape/); (B) The HNF4A potential binding sites in *KIF5B* promoter predicted by PROMO online tools (http://alggen.lsi.upc.es/cgi-bin/promo_v3/promo/promoinit.cgi?dirDB=TF_8.3). (C) The change from *MGAT3* rs34943118 T allele to C allele may alter the predicted TF-binding motif for ATF2 predicted by using HOCOMOCO-11 collection from the PERFECTOS-APE online tools; (D) The ATF2 potential binding site in *MGAT3* promoter predicted by PROMO online tools. Abbreviations: SNP, single nucleotide polymorphism; TF, Transcription Factor; PERFECTOS-APE: Predicting Regulatory Functional Effect by Approximate P-value Estimation.

**Figure S7.** Location and functional prediction of the three novel SNPs in the ENCODE project. Location and functional prediction in the ENCODE project adapted from the UCSC Genome Browser: (A) SNP *KIF5B* rs211304, (B) SNP *FMN1* rs117648907 and (C) *MGAT3* rs34943118. The H3K4Me1, H3K4Me3 and H3K27Ac tracks indicated by color density plots are associated with the enhancer and promoter regions. DNase Clusters indicated by horizontal bars show DNase hypersensitive areas. The Tnx Factor ChIP tracks indicated by horizontal bars show regions of transcription factor binding of DNA. Abbreviations: SNP, single nucleotide polymorphism; ChIP, Chromatin Immunoprecipitation; Tnx Factor ChIP, Transcription Factor ChIP-seq from ENCODE; TF, transcriptional factor.
